# Freshwater Detention by Oyster Reefs: Quantifying a Keystone Ecosystem Service

**DOI:** 10.1371/journal.pone.0167694

**Published:** 2016-12-09

**Authors:** David A. Kaplan, Maitane Olabarrieta, Peter Frederick, Arnoldo Valle-Levinson

**Affiliations:** 1 Engineering School of Sustainable Infrastructure and Environment, University of Florida, Gainesville, FL, United States of America; 2 Department of Wildlife Ecology and Conservation, University of Florida, Gainesville, FL, United States of America; Universidade de Aveiro, PORTUGAL

## Abstract

Oyster reefs provide myriad ecosystem services, including water quality improvement, fisheries and other faunal support, shoreline protection from erosion and storm surge, and economic productivity. However, their role in directing flow during non-storm conditions has been largely neglected. In regions where oyster reefs form near the mouth of estuarine rivers, they likely alter ocean-estuary exchange by acting as fresh water “dams”. We hypothesize that these reefs have the potential to detain fresh water and influence salinity over extensive areas, thus providing a “keystone” ecosystem service by supporting estuarine functions that rely on the maintenance of estuarine (i.e., brackish) conditions in the near-shore environment. In this work, we investigated the effects of shore-parallel reefs on estuarine salinity using field data and hydrodynamic modeling in a degraded reef complex in the northeastern Gulf of Mexico. Results suggested that freshwater detention by long linear chains of oyster reefs plays an important role in modulating salinities, not only in the oysters’ local environment, but over extensive estuarine areas (tens of square kilometers). Field data confirmed the presence of salinity differences between landward and seaward sides of the reef, with long-term mean salinity differences of >30% between sides. Modeled results expanded experimental findings by illustrating how oyster reefs affect the lateral and offshore extent of freshwater influence. In general, the effects of simulated reefs were most pronounced when they were highest in elevation, without gaps, and when riverine discharge was low. Taken together, these results describe a poorly documented ecosystem service provided by oyster reefs; provide an estimate of the magnitude and spatial extent of this service; and offer quantitative information to help guide future oyster reef restoration.

## Introduction

Oyster reefs provide myriad ecosystem services, including water quality improvement, fisheries and other faunal support, shoreline protection from storm surge, and economic productivity [1–3]. Oyster reefs also face a variety of anthropogenic threats, making them one of the most endangered marine habitats [4]. The most significant human-induced pressures on reefs include overfishing [5], coastal development [6], changes in hydrology and water chemistry driven by local/regional water management [7], global climate change [8], and interactions among multiple stressors [9]. Globally, estimates of oyster reef decline exceed 85% [4, 10]. Critically, even where oyster reefs remain, many survive in a degraded state relative to historical biomass and production levels [10].

Given their central role in supporting coastal resilience [11] and providing ecosystem services [1], the sustainable management of healthy oyster reefs and the restoration of degraded reefs is a primary goal of federal, state, and local coastal resource management agencies. More than $US10 million was earmarked for oyster reef restoration by the US National Oceanic and Atmospheric Administration (NOAA) in 2009 alone [10], and nearly $US40 million has been invested in oyster reef restoration in the Chesapeake Bay (MD, USA) in recent decades [12]. While this large investment is often justified in terms of the value of ecosystem services provided by oyster reefs [13], one potentially vital and economically valuable ecosystem service provided by reefs has been largely overlooked: their role in directing flow and regulating estuarine salinities during non-storm conditions.

**Fig 1:**
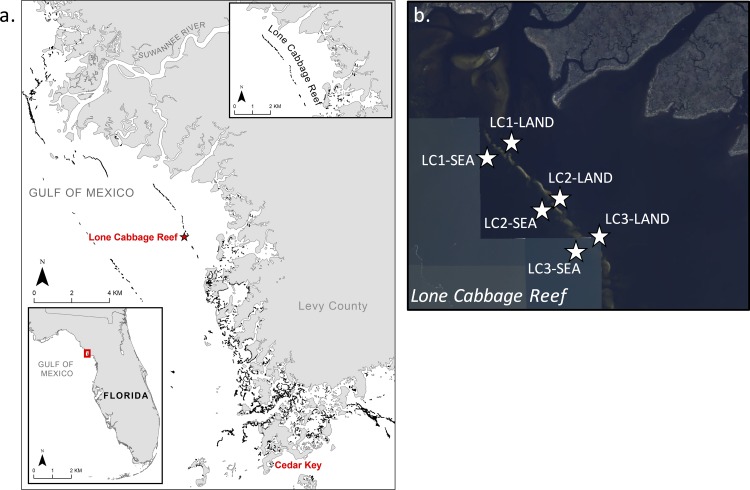
Study area and experimental setup. Salinity was measured at three paired locations on the landward (LAND) and seaward (SEA) side of the Lone Cabbage (LC) Reef, a linear reef chain located ca. 3 km from the mouth of the Suwannee River East Pass. Panel (a) reprinted from [15] under a CC BY license, with permission from BioOne, original copyright 2016. Basemap mosaic in panel (b) from the USGS National Map Viewer.

In many regions, oyster reefs form linear structures parallel to the coast and/or across the path of estuarine and riverine outflows (Fig 1), potentially altering estuary-ocean by acting as freshwater “dams”. As semi-permeable barriers, these reefs may retain fresh or brackish water in estuaries [14], a function also performed by barrier islands where they are present. We hypothesize that these reefs thus have the potential to influence salinity over areas many times greater than the reef footprint, thus providing a “keystone” ecosystem service by supporting multiple coastal functions that rely on the maintenance of estuarine (i.e., brackish) conditions in the coastal environment. Critically, in regions where shore-parallel reefs have been degraded, this service is likely lost or reduced, potentially initiating a feedback loop whereby reef loss results in decreased freshwater detention and higher estuarine salinities. Increased salinities in turn drive increased mortality of oysters via marine predators and disease, leading to eventual reef collapse [7]. In this work, we investigate the effects of shore-parallel reefs on estuarine salinity using field data and hydrodynamic modeling in a degraded reef complex in the northeastern Gulf of Mexico.

## Materials and Methods

### 2.1 Study Area

In the Big Bend region of Florida (USA), oyster reefs form km-long structures that are nearly parallel to the coast and lie wholly or partially across the mouths of the Suwannee and smaller coastal rivers and creeks (Fig 1). While extant oyster reefs in the northern and northeastern Gulf of Mexico are thought to represent some of the highest quality habitat remaining globally [4], reefs within Florida’s Big Bend have declined by 66 to 88% in the past 30 years [7], mirroring the global trend. This unique region is dominated by low-energy shoreline habitats (seagrass beds, salt marshes, and oyster reefs), has extremely low human population densities (ca. 10 people/km^2^ in the coastal counties that make up the Big Bend region, compared to a statewide average of >100 [16]), little infrastructural shoreline modification, and a large proportion of coastline under state or federal management. The overwhelming loss of oyster habitat along this largely undeveloped coastline poses a set of vexing scientific and management questions about the causes of reef decline in the region and how to best promote oyster reef conservation and restoration.

Multiple hypotheses exist to explain reef loss in the region, however recent evidence [7] suggests that reductions in freshwater discharge can trigger a cascading decline in oyster reef resilience. Low freshwater discharge leads to increased magnitude, frequency, and duration of saltwater intrusion events, which drive high oyster mortality rates due to disease and predation. Once these reefs lose their covering of living shell, they begin to break apart, spread out, and rapidly lose elevation (ca. 7 cm/yr [15]). In some locations, chains of offshore reefs become truncated and gaps between reefs enlarge [7], likely leading to further saltwater intrusion and exacerbated oyster mortality. Loss of oyster settlement substrate eventually leads to the conversion of millennia-old [17] oyster reefs to sandbars.

### 2.2 Experimental Setup

The expected effect of freshwater detention by shore-parallel oyster reefs is the maintenance of consistently lower salinities on the landward sides of reefs relative to seaward sides. To test this expectation, six monitoring stations were established to measure salinity differences between seaward (SEA) and landward (LAND) sides of the Lone Cabbage Reef, located in the Suwannee Sound near Suwannee, FL (Fig 1; 29.2576°N, 83.1023°W). Paired stations were deployed adjacent to the reef along a gradient from high to low freshwater influence, driven by outflow from the Suwannee River East Pass. Each station consisted of a conductivity-temperature-depth (CTD) sensor (CTD-Diver, Schlumberger Water Services, Tucson, AZ) logging at 15-minute intervals. Sensors were inserted into a slotted PVC housing mounted via an iron bar to a cement block. Stations were placed to keep sensors submerged at low tide (30–45 cm above the seabed) and were located between 5 and 30 m from the reef edge; the seaward reef slope was gentler than the landward side, requiring station placement at a greater distance from the reef. Maximum distance between sensors was 100 m. Data were downloaded and sensor housings were cleaned every 2–4 months. No specific permissions were required to collect these data due to the study location in coastal waters considered as state submerged lands. The study did not involve endangered or protected species. In addition, daily, tide-filtered discharge data were obtained from the USGS Gopher River station on the Suwannee River (station ID: 02323592) for comparison with salinity measurements at the reef.

Salinity data were collected from 11/2013 to 6/2015, providing ca. 200,000 15-minute salinity measurements (S1 Dataset). However, data gaps due to equipment maintenance and replacement reduced the total number of paired readings (i.e., LAND vs. SEA at a particular location) to ca. 77,000. Additionally, simultaneous data were available from all six stations for 11/2013 to 7/2014, so this period was used for comparisons among stations. Data were decimated (Lanzos filter with half power of 30 h) to tide-filtered mean daily values for these comparisons.

### 2.2 Numerical Modeling

**Fig 2:**
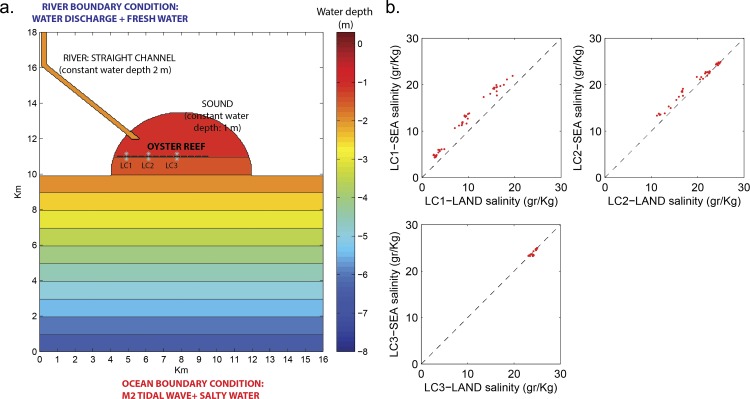
(a) ROMS model bathymetry, boundary conditions, and synthetic monitoring points (LC1, LC2, LC3). (b) Comparison of landward (LAND) and seaward (SEA) salinities between paired synthetic monitoring stations under the present reef configuration (Run 2), with different values of river discharge (5, 10 and 20 m^3^/s).

The roles of reef morphology and freshwater discharge in controlling freshwater detention were evaluated using the Regional Ocean Modeling System (ROMS). ROMS is a 3-D, free-surface, terrain-following numerical model that solves finite-difference approximations of the Reynolds-Averaged Navier-Stokes (RANS) equations using the hydrostatic and Boussinesq assumptions [18, 19] with a split-explicit time-stepping algorithm [20, 21]. A simplified inlet/sound configuration was assumed for the Suwannee Sound, consisting of a straight channel emptying into a “sound” area connected to the offshore region (Fig 2). Domain dimensions, lagoon bathymetry, and modeled reef elevations were informed by direct observations.

Simulations were performed in two steps. The first step was to consider a morphology that closely mimicked the current geometry of the Lone Cabbage Reef. The aim of this simulation was to verify that the simplified model domain captured essential features of the overall physical behavior of the Suwannee Sound and Long Cabbage Reef, rather than explicitly matching observations. After this verification, the second step was to explore ten idealized reef configurations that spanned a range of reef lengths, widths, heights, and inlet spacing (Table 1). In both simulation steps, model domains included synthetic monitoring points (Fig 2A) to emulate field sampling locations (Fig 1B), and thus compare essential features of measured and modeled results.

**Table 1:** Modeled oyster reef geometries. RL = Reef length (km), RH = Reef height (m), RW = Reef width (m) and IO = Inlet opening width (m). Note: the oyster reef is absent in Run 1, and Run 2 represents the scenario closest to the current reef geometry.

Run	RL	RH	RW	IO
1	0	-	-	-
2	5.5	1	50	50
3	5.5	0.87	50	50
4	5.5	0.75	50	50
5	5.5	1	100	50
6	5.5	1	150	50
7	5.5	1	50	100
8	5.5	1	50	150
9	5.5	1	50	0
10	8.0	1	50	50

Model cells were 50 m in the *x* (east) and *y* (north) directions, and flow structure was solved using 10 terrain-following, equidistant vertical layers. The salinity and temperature at the southern boundary were considered constant (25 g/kg and 20°C, respectively). River salinity and temperature were assumed to be 0 g/kg and 12°C, respectively. Newman boundary conditions were applied at the eastern and western boundaries. Bed friction was computed assuming the “law of the wall.” Bed-roughness lengths were chosen as 0.02 cm over sandy areas and 0.78 cm over the oyster reefs, according to [22]. Eddy viscosity was set to 0.05 m^2^/s, based on Madsen et al. [23]. To simulate the salinity ranges observed, the model boundaries were forced with a tidal wave at the southern boundary and constant fresh-water discharge of 5, 10 and 20 m^3^/s at the northern boundary. The tidal wave was defined by the four most energetic constituents in the region (semidiurnal M2 and S2 and diurnal O1 and K1). The amplitudes and phases were based on measurements at the nearest tidal gauge (Cedar Key, FL—Station ID: 8727520). The model was run for a period of 2 months for each value of freshwater discharge.

## Results

### 3.1 Field Measurements

**Fig 3:**
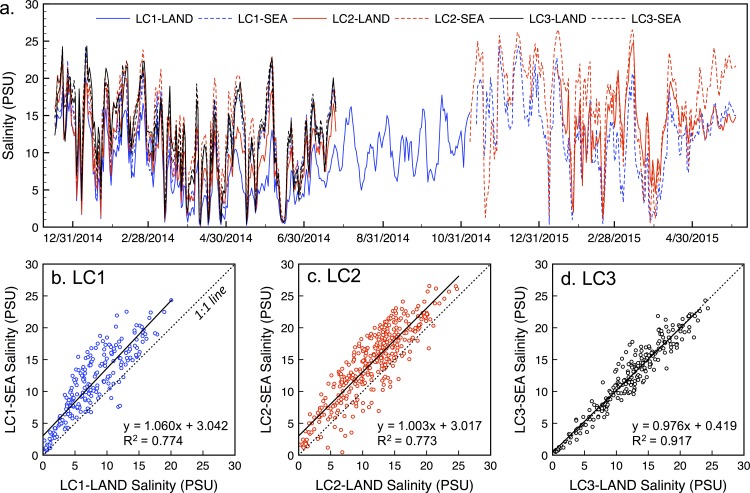
(a) Tide-filtered mean daily salinity over the 18-month period of record for the six monitoring locations in the Suwannee Sound. (b-d) Comparison of landward (LAND) and seaward (SEA) salinities between paired stations, illustrating a marked freshwater detention effect close to the mouth of the Suwannee River East Pass (LC1) that declines with distance (LC2) and becomes negligible at LC3.

Salinity at all sites was highly variable over seasons and tidal cycles, varying between ca. 0 and 27 g/kg and was generally inversely correlated with Suwannee River discharge. Tide-filtered mean daily salinity (Fig 3A) was lowest at stations closest to the mouth of the Suwannee River and increased with distance (mean±SD salinity = 9.5±5.3, 11.8±5.4, and 12.5±5.5 g/kg for sites LC1, LC2, and LC3, respectively). The largest differences between landward and seaward salinities were also observed closest to the river mouth; mean differences in daily salinity were 3.5±2.6, 3.0±2.7, and 0.1±1.6 g/kg for sites LC1, LC2, and LC3, respectively. These differences correspond to average salinity reductions of 33%, 16%, and 0% between seaward and landward stations at LC1, LC2, and LC3, respectively.

Fig 3B–3D show how these relationships varied among sites. Landward and seaward salinities were well correlated across sites, as expected given proximal sensor locations. At LC1 and LC2, we observed consistently higher seaward salinities (linear regression assuming zero intercept, with slopes of 1.36 and 1.22, respectively). At LC3, the linear regression and 1:1 lines overlap, illustrating negligible differences between landward and seaward salinities at this location. At LC1, seaward salinities were higher than landward salinities 96% of the time; this proportion declined to 89% and 51% for stations LC2 and LC3, respectively.

### 3.2 Model Simulations

Tide-filtered, mean daily salinity modeled in Run 2 (i.e., the simulation modeled with present reef conditions) was lowest closest to the river mouth and increased with distance (Fig 2B), consistent with field measurements (Fig 3A). Additionally, modeled mean daily salinity differences between landward and seaward sides of the reef corresponded to reductions of 27% and 5% at LC1 and LC2, respectively, compared with field observations of 33% and 28% reductions. Although measured and modeled values were distinct, the model captured the overall system behavior in two important ways: first, overall salinity was lowest close to the river mouth and increased with distance; second, the salinity difference between landward and seaward locations decreased with distance from the inlet. The model captured this behavior despite using simplified (vs. actual) bathymetry, constant (vs. temporally varying) Suwannee River discharge, modeled (vs. measured) tides, and neglecting wind forcing.

**Fig 4:**
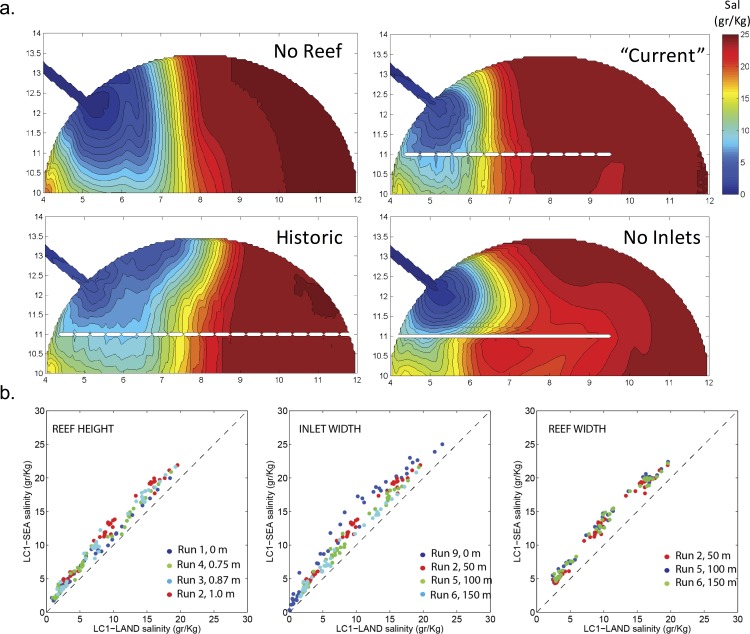
(a) Modeled daily-average subtidal salinities across four reef geometries (Runs 1, 2, 9, and 10) under freshwater discharge of 10 m^3^s^-1^. (b). Comparison of modeled landward vs. seaward salinities at LC1 illustrate effects of reef height, inlet width, and reef width under a variety of flow regimes (5, 10, and 20 m^3^s^-1^).

**Fig 5:**
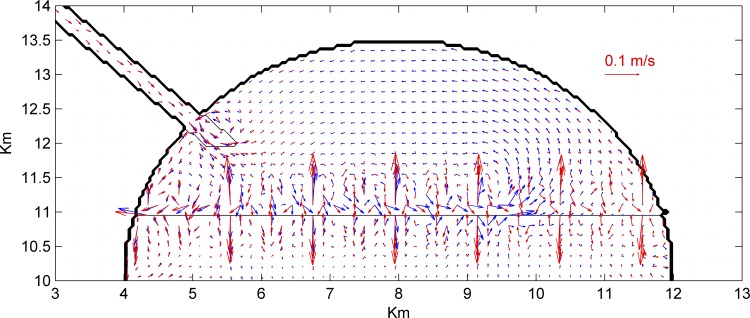
Daily-average subtidal flow velocities for Run 2 (short-reef case, blue) and Run 10 (long-reef, red), illustrating strong counter-clockwise circulation in the short-reef case that is absent when the reef fully extends across the domain.

Next, different reef geometries were considered to ascertain the role of reef length, height, and width, as well as the width of the inlet openings (Table 1). Reef lengths played a critical role on lateral salinity distribution within the sound (Fig 4A). With no reef, the freshwater plume extended further offshore and alongshore (eastward) than in runs with partial reefs. In contrast, the alongshore extension of the plume increased when the reef extended across the entire sound. This indicated that the relatively long reef maximizes freshwater detention. Distributions of M2 tidal ellipses and subtidal flows in the short- and long-reef configurations (Fig 5) showed that tidal currents became stronger through the opening in the short-reef cases, forming a counter-clockwise subtidal circulation in the sound. In the no-reef or long-reef configurations, tidally-averaged currents prevented this counterclockwise circulation, resulting in a larger region of freshwater influence. Finally, comparison of subtidal (or tidal-filtered) daily mean salinities at LC1 (Fig 4B) indicated that higher elevation and wider reefs with smaller inlets increased salinity differences between the landward and seaward sides of the reef. In all cases, salinity differences increased as freshwater discharge decreased.

## Discussion

We used field observations and numerical modeling to explore the role of oyster reefs in detaining fresh water from rivers and modifying salinities in the coastal environment. Results provide evidence that freshwater detention by oyster reefs plays a key role in modulating salinities not only in the oysters’ local environment, but over extensive coastal areas (tens of square kilometers) as a function of reef morphology. These findings have relevant implications for coastal freshwater management, oyster reef restoration, estuarine eco-hydrological modeling, and the appropriate valuation of ecosystem services provided by ecosystem engineers.

Field measurements (Section 3.1) confirmed that salinity differences exist between landward and seaward sides of the Lone Cabbage Reef over short distances (<50 m) and over both tidal and seasonal time scales (Fig 3). These effects were most pronounced closest to the mouth of the Suwannee River, where overall salinities were lowest. The Lone Cabbage Reef is relatively undegraded in this location, with the highest density of live oysters and highest elevation of all reefs in the chain. Both oyster density and elevation decline with distance from the river mouth [15]. These results reinforce the idea that healthy, higher elevation reefs have the best potential to modulate high salinities, improving the suitability of their local environment, while degraded reefs can quickly lose this function. While the reef system studied in this work is intertidal and oriented parallel to the shore (perhaps representing an extreme example of freshwater detention), it seems likely that healthy subtidal reefs may also have sufficient elevation to perform similar functions in shallow estuaries.

Numerical results (Section 3.2) expanded these observational findings by illustrating how reefs affect the lateral and offshore extent of freshwater influence. In general, freshwater detention was most evident when the reef extended completely across the sound (Fig 4A). If the reef became degraded or absent in the eastward portion of the sound, tidal currents increased while subtidal currents displayed counterclockwise recirculation within the sound. This recirculation reduced freshwater plume extension in the sound and exposed its eastern portion to higher salinities. Critically, loss of reef extent and elevation at the Lone Cabbage Reef has proceeded largely from east to west, and the mechanisms modeled here suggest that reef loss is likely to continue as the reef’s freshwater detention capacity continues to decline.

Model results also suggest that the capacity for freshwater detention within the sound increases when the reef is highest (echoing the results above), without inlets, and when discharge is low (i.e., during droughts) (Fig 4B). The presence of wide inlets along the reef reduces detention, suggesting that restoration of more complete and longer reefs may be required to provide adequate freshwater detention. The buffering effect of the reef on salinities during low flow events is of particular relevance because oyster mortality is thought to be extreme during these conditions. Low-flow events in Suwannee Sound have been observed to be increasing [7]. Taken together, the field and numerical findings presented here provide support for expanding active management efforts at degraded reefs to restore historical reef extent and elevation.

The coupling of reefs with salinities provide a potential feedback loop in which oyster reef degradation allows more saltwater intrusion during low freshwater flow events, leading to further oyster mortality and reef degradation. The strength and rates of these feedbacks depend on interactions between the rate of change of oyster reef configuration (i.e., via reef growth/deflation driven by recruitment, growth, mortality, etc.) and the salinity regime. These reciprocal feedbacks may dictate the potential for critical transitions [24] between equilibrium/thriving and collapsing/degraded reefs, but are generally neglected in existing models of oyster reef growth and decline [25–27]. For this study site, salinity measurements and the observed spatiotemporal pattern of reef loss (i.e., from east to west) and along salinity gradients [7] are consistent with increased reef vulnerability at higher salinities, while modeling results support the potential presence of a positive feedback loop of continued reef degradation after a certain threshold of loss has been exceeded.

Understanding when and where these critical transitions and stable equilibria occur will be essential for guiding successful oyster reef management and restoration and represents an important next step for advancing this work. A robust understanding of the feedbacks among freshwater flow, reef morphology, and estuarine salinity can be leveraged via active restoration to make coastal environments more resilient to sea level rise and episodic declines in freshwater discharge, particularly since oyster reefs have been shown to grow vertically at rates far in excess of sea-level rise [28]. Additionally, since healthy reefs have the potential to modulate estuarine salinities in extensive areas, direct restoration of oyster reefs can have a variety of indirect restoration impacts via the maintenance of estuarine conditions and erosion protection in nearby salt marsh, mangrove and/or seagrass beds. The results of this work may thus support the use of restored and created oyster reefs to help detain freshwater and maximize estuarine character in an era of salinizing estuaries.

Finally, oyster reefs have been shown to provide a number of important ecosystem services, including storm surge abatement, water filtration, sequestration of nutrients, stabilization of sediments, and creation of nursery habitat for fishes and invertebrates [3, 13, 29, 30]. However, their role in detaining freshwater and modulating estuarine salinities has not been considered when allocating funds for restoration, nor when assessing the monetary value of ecosystem services they provide [31]. The results presented here suggest that freshwater detention can be a critical ecosystem service provided by oyster reefs, representing a keystone service on which adjacent estuarine ecosystems rely.

## Supporting Information

S1 Dataset S1 Dataset.xlsx. 15-minute salinity, temperature, and water level data from the six monitoring stations shown in Fig 1. Data were decimated (Lanzos filter with half power of 30 h) to tide-filtered mean daily values for all subsequent analyses.
(XLSX)
